# Supplementary material for: Saccharomyces boulardii (CNCM I-745) improves intestinal damage in sepsis by remodeling intestinal flora structure
Source: Front Cell Infect Microbiol. 2025 Jul 31;15:1602792. doi: 10.3389/fcimb.2025.1602792 (PMC12350329; doi:10.3389/fcimb.2025.1602792)
Supplement: Supplementary file 1 [file DataSheet1.zip › summary/summary/6_Taxonomy_community/1_Taxonomy_profiling/1_Domain/Group/Domain_abund_top30.Group.cluster.pdf]

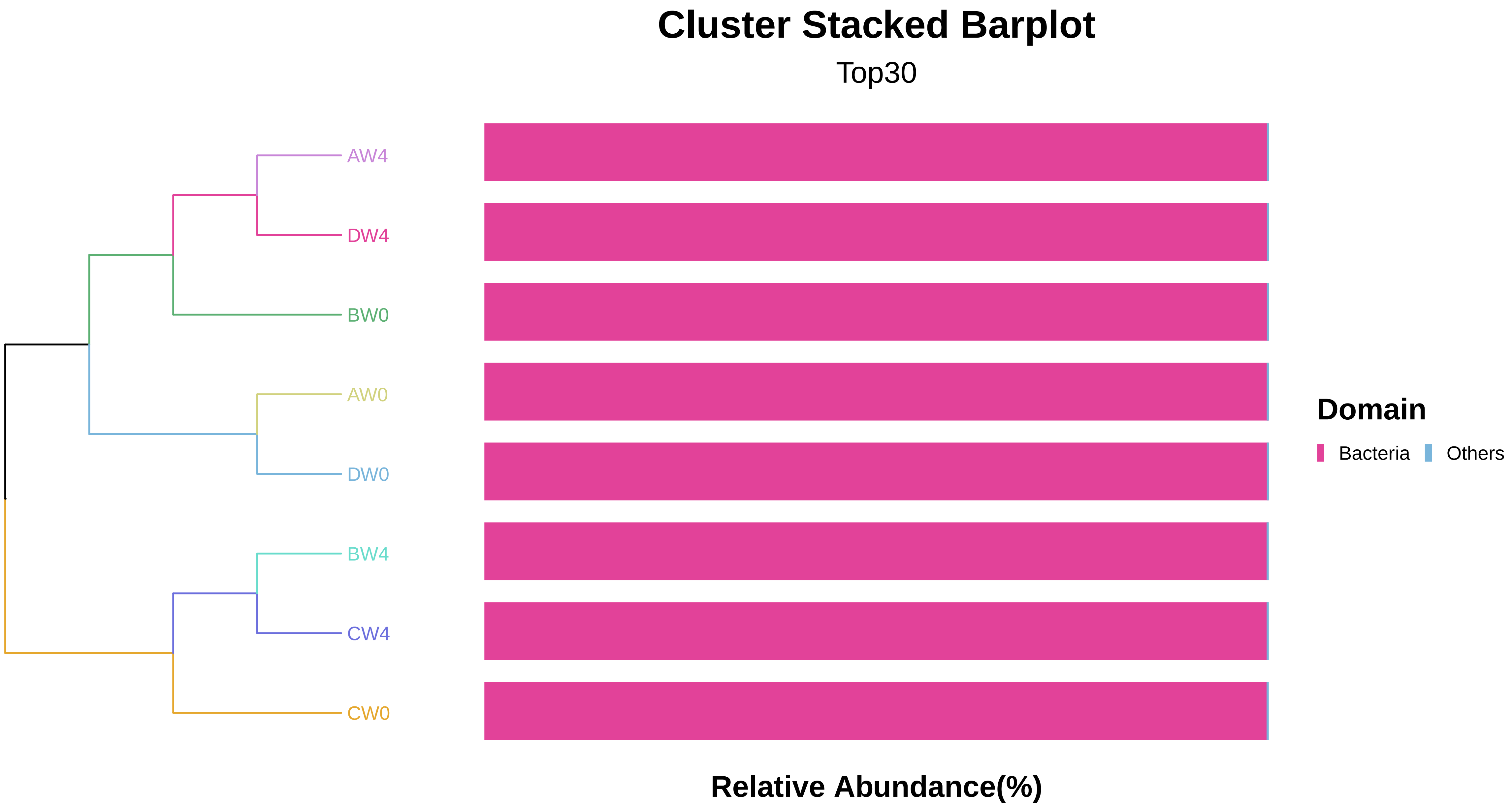
Cluster Stacked Barplot
Top30
AW4
DW4
BW0
AW0
DW0
BW4
CW4
CW0
Domain
Bacteria
Others
Relative Abundance(%)
